# Designing on-farm trials: an example with interventions to improve micronutrient status of grain crops

**DOI:** 10.1038/s44264-025-00101-0

**Published:** 2025-10-31

**Authors:** R. M. Lark, M. G. Manzeke-Kangara, J. M. Kihara, M. R. Broadley

**Affiliations:** 1 School of Bioscience, University of Nottingham, Leics, United Kingdom; 2 Rothamsted Research, Herts, United Kingdom; 3 Alliance for Bioversity and CIAT, ICIPE Duduville Campus, Nairobi, Kenya

**Keywords:** Plant sciences, Biogeochemistry, Environmental sciences

## Abstract

Design of on-farm experiments to achieve particular objectives, including statistical power, precision of estimates of treatment effects and spatial coverage, remains to be systematically studied. We assessed design options for an extended network to evaluate micronutrient-biofortification interventions for cereal crops in Ethiopia. We identified feasible designs to detect plausible treatment effects with power ≥0.8. Sufficient replication at farm-scale (where each farm is a complete block) was critical for this. To estimate the treatment mean with precision requires sufficient regional replication at the scale of farm clusters. With 250 clusters across the region the median distance from a random point in the region to an experimental site exceeded 5 km, so active steps would be needed to engage farmers, by facilitating visits to experimental sites. The approach used here could be applied more generally to design effective and efficient on-farm experimental networks.

## Introduction

In Africa, and elsewhere in what it loosely called the ‘Global South’, food supply is largely dependent on smallholder producers^1^. As well underpinning food security, the contribution of smallholder farmers is essential for achieving environmental objectives for land management, such as increasing soil carbon stocks^2^. However, it is forecast that smallholder production will decline by between 10 and 50% to 2050, over much of sub-Saharan Africa given climate change and the decline of the natural resource base, assuming ‘business as usual’ without adaptation and the take-up of agricultural innovations^3^. Along with this threat to food security, the nutritional security of populations dependent, in large part, on subsistence or other small-scale local food production, is widely undermined by inadequate micronutrient supply from staple crops. Deficiency of mineral micronutrients such as zinc (Zn) and selenium (Se) is widespread in sub-Saharan Africa, and elsewhere, with implications, *inter alia*, for child health and development^4,5^.

Agricultural interventions suitable for smallholders can address the problem of micronutrient deficiency, by improved management^6^ and crop breeding^7^. Scientists address other challenges which the smallholder faces but fundamental obstacles, including limited access to resources and inadequate technical support, prevent many farmers from taking up innovations^8^. It has been suggested that effective scientific solutions for agricultural problems require better engagement with producers, and their priorities^9^.

Chief among the strategies to improve the impact of agricultural reseaarch for smallholders is the use of on-farm experimentation to support ‘participatory’ agricultural development through a process which does not privilege technocratic outsiders at the expense of the rural community whose development is the ostensible objective of the research.

On-farm research has become increasingly popular globally. For example, Mihiretu et al.^10^ report a large on-farm experiment in marginal drylands of Ethiopia to evaluate new sorghum varieties against locally favoured ones. They concluded that on-station trials could not substitute for the on-farm evaluations, not least because of the confidence in the innovations which their performance on-farm engendered among the growers. Despite these benefits, Nyikahadzoi et al.^11^ emphasize that simply undertaking experiments on farmers’ fields may not suffice to promote agricultural innovations. They advocate *participatory action research*, led by communities. Similarly, Mapfumo et al.^3^ emphasize the importance of accounting for indigenous knowledge in research programmes. On-farm experiments, nonetheless, remain an essential component of participatory research.

The benefits of participatory research have been widely accepted, but there are concerns whether on-farm experiments necessarily sacrifice usual standards of scientific rigour, e.g.^12^. Early in the emergence of participatory methodology Chambers^13^ queried how far it is consistent with scientific methodology, whether there is a trade off, and, if so, how the balance should be struck. Gladwin et al.^14^ argued that levels of rigour normally expected in scientific research focused on the testing of hypotheses need not be eschewed. Whereas Chambers^15^ emphasised the unpredictability of the successful strategies by which farmers respond to the challenges of managing locally complex and dynamic systems, Gladwin et al.^14^ argued that this unpredictability could be overstated, and that participatory research can still embed standards of design, hypothesis testing and the scientific development of knowledge.

Conventional plot experiments at research farms provide robust information about treatment effects. They have practical advantages because plots and inputs can be carefully managed. There are also statistical advantages which follow from the history of site management, which means that sources of yield variation are understood, so blocking can be done efficiently, and quantitative information on the between-plot variation of responses can be used in power analysis to ensure that sufficient replication is done to detect effect sizes of interest and to estimate treatment effects with sufficient precision. It is necessary to address these same issues if on-farm experiments are to provide robust information.

There are examples of careful methodological practice to ensure power and efficiency for on-farm experiments.^16^ reports how farm-scale trials to measure impact of genetically modified crops on biodiversity in the UK were designed to achieve a target power, and Wuest et al.^17^ used data from on-farm uniformity trials to select statistically-efficient plot sizes for further experiments.

We agree that concerns about the statistical power of on-farm experiments are important and address them in this paper, but we think that the design of on-farm experimental networks raises new questions that are not encountered in on-station research. The first is the challenge of estimating the mean response to a treatment over a larger domain, the on-farm trial is more representative of conditions for which recommendations are needed than the station, so the estimated mean yield, or other output, is of interest, and its precision for some region is an important output and will depend on the distribution of experimental sites across that region.

The second question concerns opportunities offered by experiments at widely distributed on-farm sites. First, these allow one to map the experimental output by geostatistical interpolation, to identify variations arising from underlying environmental effects. These might imply that a treatment will be of greatest value in certain subregions, where recommendations could be subsequently targeted. Panten et al.^18^ and Bishop and Lark^19^ considered this approach to experimentation, the former in the context of variable viticultural sites and the latter in multifield trials across contrasting geological outcrops. Second, a widely-distributed network allows extensive recruitment of secondary participants in the trial through exposure of farmers to ‘parent’ experiments on the initial trial sites (referred to as parent-sites elsewhere in this paper) who are then recruited to run associated ‘child’ trials. The so-called parent and child network structure for on-farm experimentation can accelerate development and dissemination of new interventions among smallholder farmers^20^.

The study reported in this paper was prompted by interest in the possibility of addressing mineral micronutrient deficiencies by agronomic biofortification, or agrofortification, in which the element is applied to staple crops, either as a solid fertilizer or a foliar spray. In this case, we consider the micronutrients Zn and Se. Zn deficiency has high prevalence globally, including in sub-Saharan Africa^4,5^. Botoman et al.^6,21^ undertook on-station trials in Malawi which showed benefits from Zn fertilizer applied to the soil in maize plots, both with respect to crop yield and Zn concentration in the grain. There is a similar concern about Se deficiency^22^, and evidence for the effects of agrofortification on the Se concentration in maize grain^23^. A pilot on-farm experiment, conducted in western Amhara, Ethiopia, with wheat (*Triticum aestivum*) and teff (*Eragrostis tef*) crops, showed benefits from agrofortification for both these micronutrients^24^. Individual farms were treated as blocks within which fertilizer treatments were randomized in a single replication. Farms were selected in clusters of neighbouring farms distributed over three defined landscape units—foot slope, mid-slope, and hillslope—all of which were found within each cluster. In a second season a new set of farms was recruited within the same landscape units and clusters. Note that the same variety of wheat (TAY) and of teff (Kuncho) was used across the experiment^24^.

Manzeke-Kangara et al.^24^ showed that agrofortification methods can increase the concentration of Zn and Se in grain of wheat and teff, with effects of landscape position, method of application and macronutrient fertilizer management. This intervention requires further evaluation in an on-farm experimental network, comparable to those commonly encountered in the US^25^. Such a network would provide evidence for the value of the intervention in the farm environment across a wider region of Amhara region, including assessment of options for micronutrient application. It would also facilitate knowledge transfer within communities where trials are undertaken. In this paper we examine how information from the pilot trial can be used to address questions about the design of such a network, given the various options for development of on-farm experimentation which we discussed above.

We consider three specific experimental questions, each of which raises distinct methodological challenges, and so might be addressed, optimally, by a particular experimental design. The first question is concerned with whether the on-farm experimental network is large enough to provide evidence for an positive effect of agrofortification on micronutrient concentration in grain, assuming that the effect size exceeds some minimal threshold? In experimental methodology this is a matter for power analysis, power being the probability that, given a minimum effect size, a significant effect will be detected. That is to say, that a null hypothesis of no effect may be rejected on the basis of a *p*-value smaller than some threshold. We consider here the effect of the amount of replication (total number of farms recruited per cluster, and number of clusters) on statistical power.

The second question we consider is the precision with which an effect size can be estimated, considering the mean effect size over a domain of interest. The precision of the effect size estimate is important, for example, when evaluating whether the impact of agrofortification on micronutrient intake is cost-effective, comparing favourably with the impact of alternative interventions. We consider how this might be achieved over a region with clusters of farms, as in the original experiment in Amhara, distributed across the region of Amhara used in the original survey.

We next consider a highly spatially distributed network of single farms (which we call trial sites) for the two functions introduced above: the spatial prediction of treatment responses across the region, and the recruitment of secondary farms, associated with parent-sites from the designed trial. The precision of spatial predictions depends on the spatial distribution of the observations (experimental farms here), and can be calculated as the prediction error variance (kriging variance) given model parameters^19^. We considered the distance from a location in the domain to the nearest parent-sites as a measure of the quality of a design here, on the assumption that farmers are most likely to encounter a parent-site if it is closer than some maximum distance from their own farm.

## Results

### Variance components

The variability of the micronutrients of interest (log scale for Se) differs between these variables and with the crop. For Zn concentration in wheat, and the log Se concentration in teff, the short-range variation (between-plot within site) is the dominant component of variance (Table 1), although the between-site variation is comparable. The between-site variation is the dominant component for Zn concentration in teff and log Se concentration in wheat. The effective range of spatial dependence of the between-site component of variation depends on the distance parameter *ϕ* and the smoothness parameter *κ*. Supplementary Fig. 1 shows the likelihood profile used to select the value of *κ*, and Supplementary Fig. 2 shows the corresponding variogram plots. For wheat and teff Zn concentration and wheat and teff Se concentration (log), the respective effective ranges are 74, 48, 72 and 67 km, respectively.

**Table 1:** Variance parameters for each target variable, used in power and related analyses and derived from models for GeoNutrition experiment and surveys as described in section “Combining the sources of information”

Term	Wheat Zn /mg kg^−1^	Teff Zn /mg kg^−1^	Wheat Se /log mg kg^−1^	Teff Se /log mg kg^−1^
Variances				
Residual, ${\varsigma }_{{\rm{res}}}^{2}$	17.5	8.3	0.44	0.60
Farm:site, ${\varsigma }_{{\rm{farm}}}^{2}$	4.6	5.2	0.35	0.12
Site, ${\varsigma }_{{\rm{site}}}^{2}$	14.4	11.0	1.10	0.40
*ϕ*_site_	19.6	15.9	36.20	12.20
*κ*_site_	0.75	0.50	0.25	2.00

### Power to detect treatment effects

**Fig. 1:**
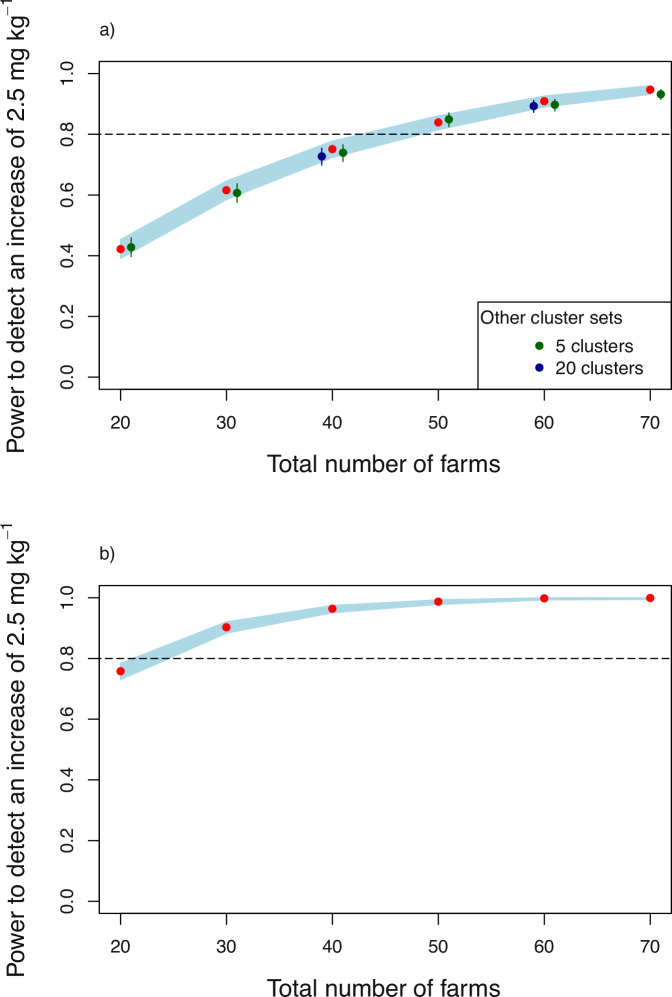
Power (red symbol) to detect a specified increase in grain Zn concentration as a function of the number of farms in 10 clusters. **a** shows power for wheat grain Zn concentration. Outcomes with different numbers of clusters are also shown, with symbols indicated in the legend. **b** shows corresponding power for trials with 10 clusters for teff grain Zn concentration. The blue band shows the 95% confidence interval.

The power to detect a target effect size on Zn concentration in wheat grain (Fig. 1a) depends on the total number of farms recruited into the experimental network, with little effect of the number of clusters over which they are distributed. For this variable the conventional target power of 0.8 to detect a difference of 2.5 mg kg^−1^ (equal to ~10% of the mean concentration in the control treatment) is achieved with ~50 farms in the experiment. Adding another 10 farms increases the power to 0.9 (Fig. 1a). With a relatively small experiment (20 farms in total) the power to detect the target effect is quite small (just over 0.5). That is to say, if the effect size specified applied but we had just 20 farms in the experiment, we would be about as likely not to detect a significant effect of the agrofortification treatment as to detect it.

For Zn concentration in teff the same required effect size of 2.5 mg kg^−1^ was specified. The target power of 0.8 can be achieved with about 25 sites, half as many as for wheat, with 50 farms the estimated power is 0.99 (Fig. 1b). Note that the experiment of just 20 farms, clearly inadequate for the wheat Zn effect, has an expected power only just below the target of 0.8 for teff grain Zn concentration, and that the power is very close to 1.0 for 60 or more farms.

**Fig. 2:**
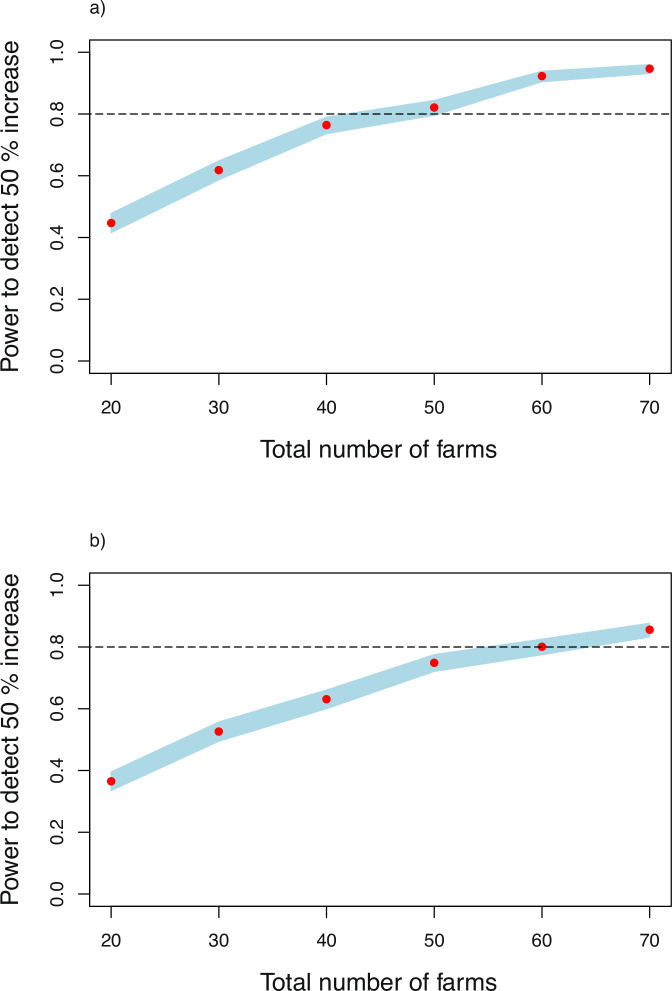
Power (red symbol) to detect a specified increase in grain Se concentration as a function of the number of farms (10 clusters). **a** shows power for wheat grain Se concentration and Fig. 2**b** shows corresponding power for teff grain Se concentration, The blue band shows the 95% confidence interval.

As noted in the Methods section, the statistics for Se concentrations in both grains were analysed on the log scale (natural logarithms to base *e*). We first considered the task of detecting a 10% increase in Se concentration, equivalent to an effect size of log(1.1) on the log scale. However, this effect is small relative to the magnitude of the random variation in grain Se, and the power to detect this effect for Se concentration in teff grain was only 0.14 with 70 farms. We also considered the challenge of detecting a rather larger effect of the biofortification, that it increases grain Se concentration by 50%—an effect size of log(1.5) on the log scale. Figure 2 shows that detecting this effect size with power of 0.8 requires 50 and 65 farms for wheat (Fig. 2a) and teff (Fig. 2b), respectively.

Note that, while there was a marked difference in power between experiments with the same number of farms for Zn concentration in grain of wheat and of teff, the results are much more similar for Se in the two crops, with power to detect a 50% increase in concentration close to 0.4 with 20 farms, and not approaching 1.0 for either grain within the range of experiment sizes considered here.

### Precision of estimates of a regional treatment mean

**Fig. 3:**
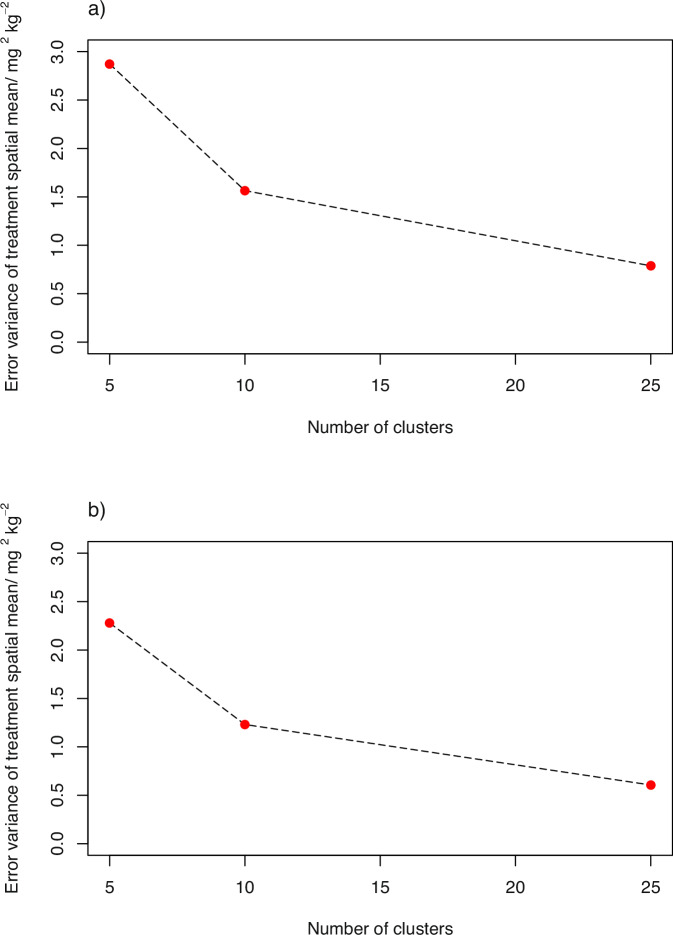
Estimation variance of the mean Zn concentration in grain over the domain of interest as a function of the number of experimentalclusters, with 50 farms in 5, 10 or 25 clusters. This is shown for (**a**) wheat grain Zn concentration and (**b**) teff grain Zn concentration.

Results for the variance with which the treatment mean (spatial mean across the domain) is estimated for grain Zn concentration from 50 farms at 5, 10 or 25 clusters, are shown in Fig. 3. Note that increasing the number of clusters from 5 to 10 has a substantial effects on the error variance for the regional mean for both teff and wheat, there is a smaller reduction in the error variance on a further increase of the number of clusters to 25. Plots such as this, therefore, allow one to select the number of clusters required if an effect size estimate is required as well as an inference about its significance.

### Precision of interpolated grain micronutrient concentration, and distance from locations in the domain to the nearest parent-site

**Fig. 4:**
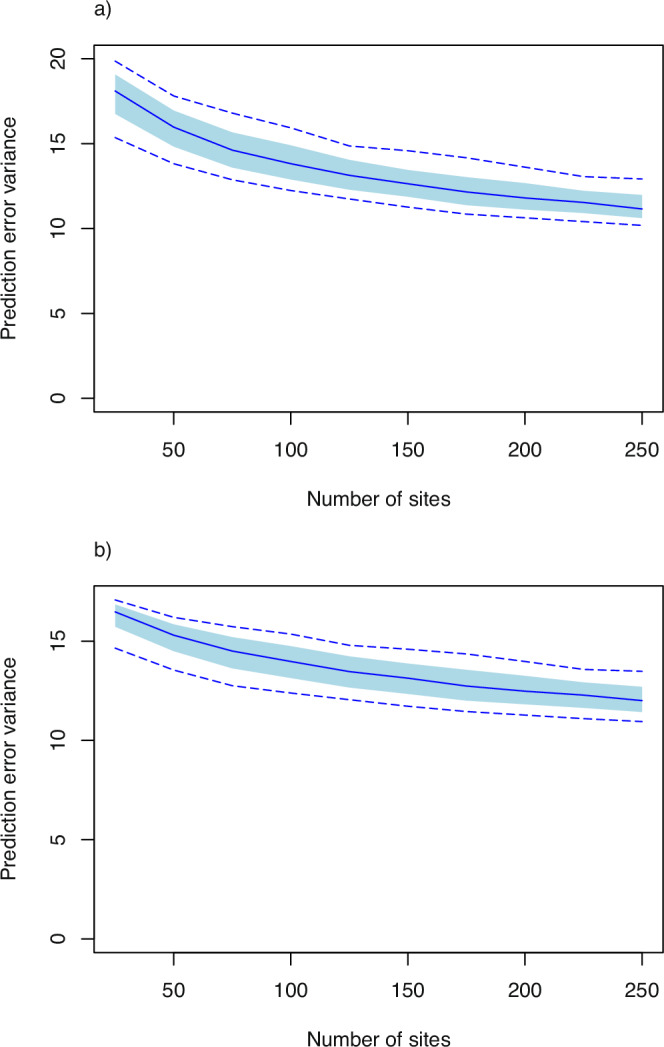
Prediction error variance of farm-scale treatment effect by ordinary kriging from spatially balanced and spread sets of parent-sites, asa function of the number of these sites. This is shown for Zn concentration in grain of (**a**) wheat and (**b**) teff.

If the number of parent-sites within a fixed region, and so the sample density, is increased a priori, this is expected to reduce the prediction error variance for farm-scale mean responses to a treatment obtained by spatial interpolation from observations at the parent-sites. Exactly how this occurs depends on the spatial variance parameters in Table 1, and the distribution of candidate sites across the region, and the form of this effect for the examples here is given in Fig. 4. As the sample density increases so the prediction error variance of the farm-scale mean Zn concentration in wheat grain (Fig. 4a) and teff grain (Fig. 4b) at an unsampled location declines, that is to say a more precise estimate is obtained. Note that the reduction in the prediction error variance per additional site declines somewhat as the total sample size increases, so that there is a smaller reduction in error variance on increasing the number of sites from 200 to 250 than there is on increasing from 50 to 100. This effect is larger for grain Zn concentration in wheat than it is in teff.

**Fig. 5:**
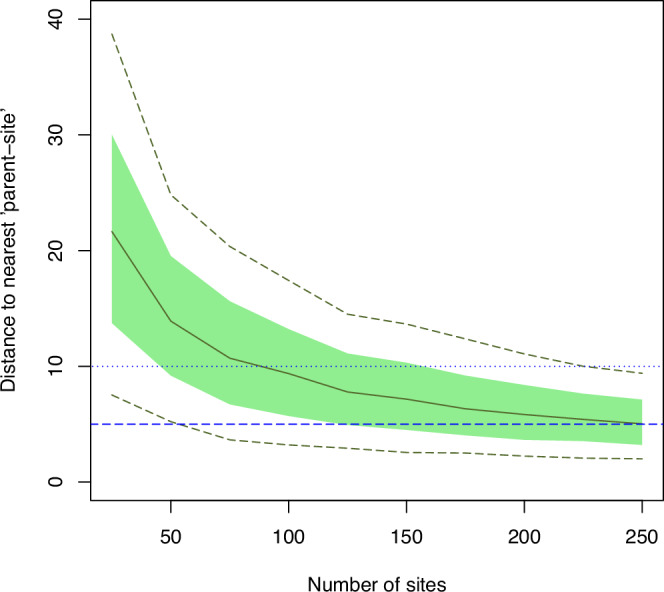
Distances (km) to nearest neighbouring parent-site as a function of parent-site number in the Amhara domain. The solid green line is the median distance, the green polygon spans the first and third quartile, and the dashed green lines show the first and 9th decile. The horizontal dotted blue line is at 10 km, the dashed blue line is at 5 km.

It is clear, intuitively, that distributing more experimental parent-sites around a fixed region will also reduce the typical distance from a particular smallholder’s farm, not included in the trial, to the nearest parent-site where they can observe the experiment. The simulations undertaken here allow us to quantify this effect. Figure 5 shows this for experimental parent-sites, selected within the Amhara domain by spatially balanced sampling. For any given number of parent-sites the solid green line shows the median distance from a site within the domain to the nearest parent-site, the dashed lines show the first and 9th decile (and so bracket the range of distances for 80% of locations in the domain) and the solid green region is bounded by the first and third quartiles (and so brackets 50% of locations in the domain). The figure shows that, if we want at least half of potential ‘child’ trial sites to be within 10 km of the nearest parent-site in Amhara region, then more than 75 parent-sites are required. To get better coverage, with at least 75% of sites in a domain within 10 km of a parent-site requires about 150 sites. If it were thought that a parent site is unlikely to recruit farmers who are more than 5 km away, then the figure shows that approaching 250 sites are needed to achieve this for 50% of locations in the domain.

As with the prediction error variance, the reduction of the distance from a smallholder to the nearest parent-site on addition of an extra site declines as the total sample size increases. Thus, on the basis of this criterion only, we might consider that the benefit from having 250 sites rather than 200 does not justify the additional cost while the benefit from having 100 rather than 50 is substantial.

## Discussion

The variance model (Table 1) used in this study is specific to the Amhara region, and a similar pilot study would be needed if an on-farm network were to be set up elsewhere. However, the approach which we outline shows how existing trial and survey data can be assimilated to address design questions. As noted the variables of interest here show spatial dependence over distances between about 50 and 80 km. This implies that there may be substantial differences in the micronutrient concentrations of grains between substantial sub-regions of Amhara, and so obtaining locally-predicted outcomes of a trial to support site-specific advice would be more efficient than using a single recommended intervention across Amhara.

The results (section “Power to detect treatment effects”) show that power to detect an effect depends on the number of farms, and changes little if this remains constant but the farms are distributed between different numbers of clusters. This result is not unexpected because the treatment comparisons are made within balanced blocks (farms) and so the variation between these is immaterial to the inference about treatment effects. If, however, incomplete blocks were used because more treatments were required than can be accommodated on a single farm then some attempt should be made to achieve within-cluster balance, and the numbers of farms per cluster would affect the power to detect effects of interest partly estimated from between-farm comparisons.

In the case of Amhara and the responses of interest, the results show that, on considerations of power alone, an on-farm network for teff Zn agronomic biofortification could be markedly smaller than one for wheat (50 farms are required to achieve the target power for wheat, and half as many for teff). If power to detect effects were the only consideration, then this could provide a basis for efficiently dividing a total set of participating farms between those allocated trials on teff and those allocated trials on wheat. However, this might compromise other objectives of the on-farm research. First, it would reduce the level of participation by individual farmers with interests in both crops so that the network would not benefit from their experience, and the communication of experimental findings to the wider smallholder community would be impaired. It would also reduce the efficacy of the network for other statistical objectives, as we now consider. The power considerations are not irrelevant, however. They might show that an affordable trial is not sufficiently well-powered to detect effects of interest, which could inform a decision to focus on fewer treatments.

The regional mean response to a treatment (section “Precision of estimates of a regional treatment mean”) is an important variable to estimate, as it allows assessments to be made of the impact of an intervention at regional scale which can inform the use of limited resources by policy makers and extension services. Because the spatial variation of grain Zn concentration, without any intervention, is modelled in these studies as spatially dependent, the number of clusters (when these are selected according to a spatially balanced design) does influence the precision of the estimate, and is improved when the network as a whole achieves good spatial coverage of the region. While the power of a network to detect an effect is important, it cannot be the only consideration, and the precision of the estimate of the effect size may also affect important practical considerations. This will have implications for network design because the distance travelled to visit all experimental farms will increase more, on average, on the addition of a cluster to the network than on the addition of an equivalent number of farms to existing clusters.

Spatial predictions, at farm scale, of the response to a treatment may be useful for making site-specific decisions on the adoption of an intervention, and may be supported by data from a spatially distributed experimental on-farm network. As found in the results (section “Precision of interpolated grain micronutrient concentration, and distance from locations in the domain to the nearest parent-site”) the prediction error variance declines with increasing numbers of sites (i.e. the precision increases).

What constitutes sufficient precision of this estimate is difficult to state outside a specific context^26^. The expected cost from making decisions from uncertain spatial predictions depends on the options available and the costs of inputs and opportunity costs from not using them. It is possible, however, to elicit target precisions in particular circumstances (e.g.^27^), and output such as Fig. 4 may be used to select the number of sites to achieve this target.

What is notable over this range of potential network sizes, given the range of spatial dependence of grain Zn concentration, is that the precision of the estimate is relatively insensitive to the size of the network. For example, reduction of the network from 250 to 125 increases the median prediction error variance for Zn concentration in wheat grain over the domain from just over 11 (units are mg^2^kg^−2^) to just over 13. The corresponding increase in the 95% confidence interval is from ±6.7 mg kg^−1^ to ±7.3 mg kg^−1^. It is unlikely that this relatively small difference in absolute precision would itself justify the costs of the larger network, although better coverage also improves the estimate of the regional mean (see above) and accessibility to smallholders in the region (below).

The distance from a smallholder farm to its nearest neighbour in the network (Fig. 5) shows rather greater sensitivity to the number of sites than does the prediction error variance for responses. This consideration is significant in the design of participatory research because of the wish to recruit additional participants, or at least to ensure that as many smallholders as possible are exposed to the interventions under investigation and have a chance to interact with the research. The curves in Fig. 5 could be used to consider the trade-off between coverage of the network and the costs of recruiting and servicing the experiments, and of additional steps to boost exposure to the parent-sites in the trial such as organizing transport to farmer events.

In summary, with sufficient replication at farm level in complete blocks, regardless of the number of discrete sites over which those plots are distributed, sufficient power can be attained to produce evidence for treatment effects in on-farm experiments as with on-station ones. However, the potential of on-farm experimentation for improving the efficacy of agricultural research goes beyond this. Information about expected treatment mean responses (e.g. yield of a new variety) in farm conditions is important for economic and policy decisions, and requires sufficient spatial replication across a heterogeneous domain if it is to be sufficiently precise to support decision making. Estimation of local response values by geostatistical modelling could also be useful particularly, for example, where effects of a treatment are additive or interact with environmental variation. It is known, for example, that the concentration of Zn in the grain of a wheat variety bred to accumulate this mineral also depends on local soil conditions^7^. To map such effects requires sufficient on-farm sites, distributed to achieve spatial coverage to ensure that prediction error variances are acceptable. Spatial coverage with on-farm trials is also needed for their reach in the community, both to recruit additional participants and to disseminate research findings. With information from pilot studies and surveys it is possible to make rational decisions about design, considering different objectives, their trade-offs and the costs and logistical challenges of implementation.

For example, if 50 farms are used in a trial, distributed in pairs at 25 sites across the domain of the Amhara survey, then the results in Fig. 1 suggest that this would be sufficient to ensure power of 0.8 to detect an increase of Zn concentration in wheat or teff grain of 2.5 mg kg^−1^ resulting from an intervention such as agrofortification. It would also suffice to ensure that the regional mean concentration of Zn in these grains can be estimated with a prediction error variance of less than 1.0 mg^2 ^kg^−2^ (Fig. 3). The median prediction error variance for a farm-scale prediction of the concentration in wheat grain would exceed 15 mg^2^ kg^−2^ (Fig. 4) so the 95% confidence interval would be ±7.7 mg kg^−1^. The scope to increase this precision is somewhat limited (unless additional predictive covariates are found), increasing sample size to 150 sites would reduce the width of the confidence interval by about 10%, but 75% of non-trial farms would be within 10 km of an on-farm experimental site. If 25 sites were used, on the basis of the considerations above, most non-trial farm sites in the domain would be more than 10 km from their nearest trial site and 25% would be further than 20 km (Fig. 5). This is unlikely to support all the objectives of a participatory network, so it would be necessary either to facilitate travel to experimental sites for smallholders outside the network, or to improve its spatial coverage.

The objectives of an on-farm network must therefore be clear, because the size of an on-farm experimental network and the distribution of total numbers of farms between sites will affect the costs of recruiting, servicing and administering that network. We would expect the marginal cost of one more farm in an existing site to be substantially smaller than the marginal cost of adding a new site, as the latter will require a new recruitment campaign, recruitment of local leaders and travel costs to the site for inspections, data collection etc. In general we may find an optimal solution to this problem if the marginal costs or their ratio can be specified, the maximum acceptable error variances for the regional mean responses and for farms-scale spatial prediction can also be stated, and constraints on particular quantiles of distance from a smallholder to a trial site may be identified. This latter problem may require further work with stakeholders, along the lines of that done by Chagumaira et al.^26^ in connection with spatial information on micronutrient concentration in crops.

To conclude there are different considerations in the design of a participatory on-farm research network which will constrain both its costs and its effectiveness. This study has shown how information from surveys and from pilot trials can be used to compute relationships between quality measures for a network and its structure and size. Further work is needed on how stakeholders can be helped to use this information to make decisions on the tradeoffs between costs and these objectives.

Whilst this study has focused on one particular intervention (agronomic biofortification) to address a particular problem (micronutrient deficiency), the methods which are presented could be used across a range of problems where participatory on-farm research is required. For example, it could be used to study yield responses to conservation agriculture interventions, aimed to make smallholder production ‘climate smart’^3^. The approach could be particularly useful when the estimation of landscape-scale outcomes, such as changes in soil organic carbon status under alternative management systems, is of particular policy relevance^2^.

## Methods

### GeoNutrition on-farm experiments

A detailed account of the experiments used to provide information of this study is given by Manzeke-Kangara et al.^24^ for details, including the statistical model used. This was a linear mixed model with nested random effects for site of variance ${\sigma }_{{\rm{site}}}^{2}$, between-farm within-site with variance ${\sigma }_{{\rm{farm}}}^{2}$, and a residual of variance ${\sigma }_{{\rm{res}}}^{2}$, crossed with a random effect for the cropping year of variance ${\sigma }_{{\rm{year}}}^{2}$.

Manzeke-Kangara et al.^24^ undertook exploratory data analysis of the marginal residuals of their model-fitting to these experimental data to decide whether they could be analysed on the original scale of measurement (mg kg^−1^) consistently with the assumptions of the linear mixed model. For both crops the grain Zn concentration could be analysed on these units, but the Se concentration was transformed to natural logarithms. All analyses reported are on these units.

The estimated variance components for these random effects are presented in Supplementary Table 1, along with 95% confidence intervals which were obtained with the confint.merMod function from the lme4 package for the R platform. This finds a confidence interval by profile likelihood on the model parameters^28^.

For comparison with the spatial model from the GeoNutrition survey, we examined distances between farms within the experimental sites. The mean distance was 0.7 km, with a median of 0.5 km, a first quartile of 0.3 km and a third quartile of 1.0 km.

### GeoNutrition survey

The GeoNutrition survey and data are described by Gashu et al.^29,30^ and Kumssa et al.^31^. For purposes of this study we consider data from the survey on Zn concentration in grain of wheat and teff, and Se concentrations in the same grains. We fitted linear mixed models to these data. For concentration of Se in teff there was a marked west-to-east increase, which was modelled as a linear trend. For all other variables the only fixed effect was a constant mean. The random effects of the model comprised a spatially-correlated Gaussian random field, and an independent and identically distributed Gaussian residual. To make the assumption that the data were a realization of a Gaussian random field plausible it was necessary to transform the data on Se concentrations to natural logarithms. A transformation was not required for the data on Zn concentrations.

These models were fitted with the variofit function in the geoR package^32^. A Matérn spatial correlation function was specified for the spatially-dependent random effect, and, following Diggle and Ribeiro^33^, the smoothness parameter, *κ*, was estimated by calculating the profile likelihood over a number of values. The geostatistical model parameters, presented in Supplementary Table 2, describe a spatially correlated random effect among the survey observations (variance ${\sigma }_{1}^{2}$ and with spatial autocorrelation described by the parameters *κ* and *ϕ*), and an independent and identically distributed random effect (variance ${\sigma }_{1}^{2}$) which characterises short-range variability and measurement error.

An example profile likelihood plot, and plots of the variance models fitted to the survey data for Zn and (log-transformed) Se concentrations in wheat and teff grain and shown in Supplementary Figs. 1 and 2.

### Combining the sources of information

For the analyses undertaken in this study we require, for each variable (micronutrient in a particular grain), values of a spatially-correlated between-site variance component, which we denote by ${\varsigma }_{{\rm{site}}}^{2}$, with a Matérn smoothness and distance parameter, specified here by *k*_site_ and *φ*_site_ respectively. We also require a between-farm within-site variance ${\varsigma }_{{\rm{farm}}}^{2}$ and a residual variance term ${\varsigma }_{{\rm{res}}}^{2}$. In this section we discuss how these values were selected, based on models for the GeoNutrition survey and experimental data.

Both the experiment and the survey used data on micronutrient concentration in grains from a composite sample. In the survey the composite was formed from within a 100-m^2^ (0.01-ha) circular plot. In the experiment each measurement was based on a composite sample from within an experimental plot of area 25 m^2^. The support of each sample set, that is to say the size and distribution of the aliquots which make up an individual grain sample, are therefore not identical but are very similar, particularly relative to the scale of the Amhara region. The same analytical methods were used to obtain the values of micronutrient concentrations from each set of samples. On this basis, the two sets of statistics are comparable.

To address design questions we require a random effects model for grain micronutrient concentrations with a site, between-farm and between-plot (residual) component. Ideally the site component can be modelled as a spatially-dependent random function with an autocorrelation function which allows the computation of a between-site covariance matrix for any spatial distribution of sites. We restrict our analysis to consideration of effects observed within a single season, not least because the between year variance component is estimated from the two seasons of data with considerable uncertainty.

The residual variance terms reported in Table 1 comprise between-plot within farm variance and the variance of analytical error. The uncorrelated variance (${\sigma }_{0}^{2}$) in the geostatistical model also includes analytical error, and variance correlated at scales shorter than the shortest interval among sample points in the survey. As the survey included close-paired sites over 500 m, which is large relative to between-plot distances, but small relative to the effective range of the spatial models (Supplementary Table 2), we may expect these two variance terms to be approximately equal. This is born out by a comparison between Supplementary Tables 1 and 2. For this reason the larger of the two terms was treated as a conservative value for the residual variance, ${\varsigma }_{{\rm{res}}}^{2}$, to use in the model for power analyses.

In principle the between-site variation should be characterized by the distance parameters and correlated variance of the geostatistical model regularized to a site support. However, the within-site differences are two orders of magnitude smaller than the effective range of the variograms as presented in Supplementary Table 2, and so the difference between the regularized and unregularized variograms will be very small. It is therefore slightly conservative to treat ${\sigma }_{1}^{2}$ as corresponding to the between-site variance component ${\varsigma }_{{\rm{site}}}^{2}$, with spatial parameters *k*_site_ and *φ*_site_ set to the values fitted to the survey data (Supplementary Table 2). As the direct estimate of the between-site variance from the experimental data has considerable uncertainty (due to the small number of sites), this value is ignored. The between-farm within site variance ${\varsigma }_{{\rm{farm}}}^{2}$ was taken directly from the estimate provided by the experimental data, ${\sigma }_{{\rm{farm}}}^{2}$ in Supplementary Table 1. The variance terms used for the investigation of experimental designs for each variable are tabulated below.

### Designs and quality measures

The power of an experiment to detect a specified effect size is the probability that the effect would be declared significant with *p* less than some specified threshold (e.g. 0.05). A threshold power of 0.8 is often specified. We started by considering a fixed number of sites (10) and different numbers of farms per site (2, 3, .., 7). The site locations were selected by spatially balanced random sampling with spread^34^ from locations within the sampling frame for the original GeoNutrition survey of Amhara^29^. This sampling was done with the BalancedSampling library for the R platform^35^.

A covariance matrix for plot-level responses was specified for each design under consideration, using the variance parameters in Table 1 (see section “Combining the sources of information”, and with two treatments, a control and an agrofortification treatment with a specified difference in the means. For the Zn effect size we specified an increase of 2.5 mg kg^−1^ in grain Zn for both wheat and teff. This was equivalent to about 10% of the control mean concentration of Zn in wheat, and about 8% of the control mean for teff, in the GeoNutrition experiments^24^.

Because the data on grain Se for both wheat and teff (experiment and survey) were modelled on a log-scale due to the distribution of the residuals, a power analysis cannot be undertaken for a linear effect (e.g. an addition of 2.5 mg kg^−1^). Rather, one can undertake the power analysis for a specified proportional increase of *x*%. On the log scale this is equivalent to an increase of $\log (1+x/100)$. For grain Se we considered an effect size of $\log 1.1$ and of $\log 1.5$ on the log scale, i.e. a 10% and 50% increase respectively. The latter was achieved by improved macronutrient fertilization of wheat (not agrofortification) in the GeoNutrition trial.

A realization of this model was generated with the mvrnorm function from the MASS package for the R platform^36^. The simulated data were then analysed with a linear mixed model fitted with the lme function from the nlme package^37^. If the *p*-value for the null hypothesis of zero treatment effect was smaller than 0.05 then the null hypothesis was rejected. The proportion of rejections over 1000 realizations of the design was computed, and the 95% confidence interval for this estimate of power was obtained with the method of Blaker^38^ as provided in the PropCIs package for the R platform^39^.

In the case of Zn concentration in wheat grain, a number of additional cases were then simulated with either 5 sites or 20 sites, and total number of farms equal to one or more cases of the original series. This was done for illustrative purposes, the distribution of farms among different numbers of sites will not affect power systematically because, in effect, the farms are blocks.

Our second possible objective of an on-farm experimental network is to provide a domain-wide estimate of the treatment mean. For some specified spatial distribution of farms the variance of this estimate can be computed directly from terms of the variance model. This was done for a fixed total number of farms (50) distributed between 5, 10 or 25 sites. The domain was defined as the sampling frame for the original GeoNutrition survey of Amhara^29^. For a set of proposed sample sites the variance of the estimate of the domain mean was obtained with the following equation, as used, for example, by Lark et al.^40^,

1 $${\sigma }_{{\rm{m}}}^{2}=\frac{2}{n}\mathop{\sum }\limits_{i=1}^{n}\bar{\gamma }\left({{\bf{x}}}_{i},{\mathcal{B}}\right)-\frac{1}{{n}^{2}}\mathop{\sum }\limits_{i=1}^{n}\mathop{\sum }\limits_{j=1}^{n}\gamma \left({{\bf{x}}}_{i}-{{\bf{x}}}_{j}\right)-\bar{\gamma }\left({\mathcal{B}},{\mathcal{B}}\right),$$

where **x**_*i*_ is a vector that denotes the location of the *i*th out of *n* sample sites, and $\gamma \left({\bf{h}}\right)$ is the variogram of the random variation of micronutrient concentration about a treatment mean, specified in this case by the following function, with terms taken from Table 1,

2 $$\begin{array}{l}\gamma \left({\bf{h}}\right)={\varsigma }_{{\rm{res}}}^{2}+{\varsigma }_{{\rm{farm}}}^{2}+{\varsigma }_{{\rm{site}}}^{2}\left\{1-\frac{1}{{2}^{{k}_{{\rm{site}}}-1}\Gamma ({k}_{{\rm{site}}})}{\left(\frac{| {\bf{h}}| }{{\varphi }_{{\rm{site}}}}\right)}^{{k}_{{\rm{site}}}}{K}_{{k}_{{\rm{site}}}}\left(\frac{| {\bf{h}}| }{{\varphi }_{{\rm{site}}}}\right)\right\},\,| {\bf{h}}| =0\\\qquad\quad =\,0,\,| {\bf{h}}| > 0,\end{array}$$

where Γ( ⋅ ) denotes the Gamma function and ${K}_{{k}_{{\rm{site}}}}(\cdot )$ is a modified Bessel function. The term in Eq. (2) inside the large braces is 1 − the Matérn spatial correlation function.

The symbol ${\mathcal{B}}$ denotes the domain to be sampled,

3 $$\bar{\gamma }\left({{\bf{x}}}_{i},{\mathcal{B}}\right)\,=\,{\int}_{{{\bf{x}}}_{k}\in {\mathcal{B}}}\gamma \left({{\bf{x}}}_{i}-{{\bf{x}}}_{k}\right)\,{\rm{d}}{{\bf{x}}}_{k},$$

and

4 $$\bar{\gamma }\left({\mathcal{B}},{\mathcal{B}}\right)\,=\,{\int}_{{{\bf{x}}}_{k}\in {\mathcal{B}}}{\int}_{{{\bf{x}}}_{l}\in {\mathcal{B}}}\gamma \left({{\bf{x}}}_{k}-{{\bf{x}}}_{l}\right)\,{\rm{d}}{{\bf{x}}}_{l}\,{\rm{d}}{{\bf{x}}}_{k},$$

where the integrals are over the two-dimensional space of ${\mathcal{B}}$. The integrals are evaluated numerically.

This quality measure was computed only for the data on grain Zn concentration. It is possible to back-transform log-transformed variables to original units with a change of support to the domain mean, but generalized quality measures for those quantities cannot usefully be specified just from the terms of the variance model. This precludes using the model terms for log-transformed Se concentrations in grain. The approaches used here for the Zn data could, in principle, be extended to variables such as Se on a log scale, but this is a topic for further research, based perhaps on the approach of Lark and Lapworth^41^.

A third possible consideration in network design is the precision of interpolated treatment mean Zn concentrations in grain from a network of parent-sites, and distribution of distances from a farm outside the network to the nearest neighbouring farm in the network. For some given spatial distribution of sample points, and a specified variogram model, the prediction error variance of a kriged interpolation at an unsampled site may be computed^42^. Here, we assumed that a block-kriging prediction at farm scale is made and the prediction error variance was computed using the variogram in Eq. (2) and Eq. (9.24) from Webster and Lark^42^.

Again, this calculation was done only for data on Zn concentration because the computation of the block kriging variance for a prediction on a log scale cannot be done without implausible assumptions^43^.

For some specified number, *n*_m_, of parent-sites *n*_m_ locations were selected from the Amhara survey domain by spatially balanced random sampling with spread, as described in section “Designs and quality measures”. The kriging variance for mean Zn concentration in wheat or teff grain under a specific treatment was computed, as described above, at 5000 locations sampled from the Amhara survey domain, again by spatially balanced random sampling with spread. In addition, for each of these sample locations the distance to the nearest parent-site was computed.

## Supplementary information

Supplementary Information

## Data Availability

The experimental data ^24^ used in this study are currently available on request from the MGM-K. Meta-data associated with these data are available at 10.23637/rothamsted.98y40 Open access to the data will be available at https://harvestirr.rothamsted.ac.uk/ once they are published. The GeoNutrition survey data are available at 10.6084/m9.figshare.15911973 and readers are referred to Kumssa et al.^32^ for more information. The power and variance computations were done with bespoke R code which will be provided by the corresponding author on request.

## Code availability

The power and variance computations were done with bespoke R code which will be provided by the corresponding author on request.
