# Supplementary material for: Designing on-farm trials: an example with interventions to improve micronutrient status of grain crops
Source: NPJ Sustain Agric. 2025 Oct 31;3(1):58. doi: 10.1038/s44264-025-00101-0 (PMC12578627; doi:10.1038/s44264-025-00101-0)
Supplement: Supplementary file 1 — Supplementary Information [file 44264_2025_101_MOESM1_ESM.pdf]

Supplementary Material

Supplementary Table 1 Variance components, with 95% confidence interval from analysis of the GeoNutrition experiments reported by Manzeke-Kangara et al. (2023), reference 24 in the paper.

Variable	Variance component and 95% confidence interval (latter in braces)			
	Site; σ^2_{site}	Farm:Site; σ^2_{farm}	Residual; σ^2_{res}	Year; σ^2_{year}
Wheat Zn /mg kg^{-1}	18.06 {1.92, 174.79}	4.57 {1.54,7.94}	17.54 {13.94,20.44}	1.93 {0.06,55.49}
Teff Zn /mg kg^{-1}	1.23 {0.00, 26.83}	5.24 {3.33, 7.83}	3.08 {2.46, 3.52}	5.20 {0.47, 54.90}
Wheat Se /log mg kg^{-1}	0.03 {0.00, 0.60}	0.35 {0.18, 0.61}	0.44 {0.35, 0.55}	0.05 {0.00, 0.71}
Teff Zn /log mg kg^{-1}	0.02 {0.00, 0.23}	0.12 {0.02, 0.22}	0.60 {0.48, 0.72}	0.00 {0.00, 0.07}

Supplementary Table 2 Geostatistical model parameters from analysis of micronutrients in grain from the GeoNutrition survey of Amhara region

Variable	Model parameter					
	σ_0^2	σ_1^2	ϕ/km	κ	Effective range /km	Median SSEP [1]
Wheat Zn /mg kg^{-1}	15.9	14.4	19.6	0.75	70	0.46
Teff Zn /mg kg^{-1}	8.3	11.0	15.9	0.50	48	0.47
wheat Se /log mg kg^{-1}	0.4	1.1	36.2	0.25	80	0.39
Teff Zn /log mg kg^{-1}	0.5	0.4	12.2	2.00	67	0.40

[1]95% confidence interval is {0.26,0.65} for wheat data and {0.27,0.64} for teff

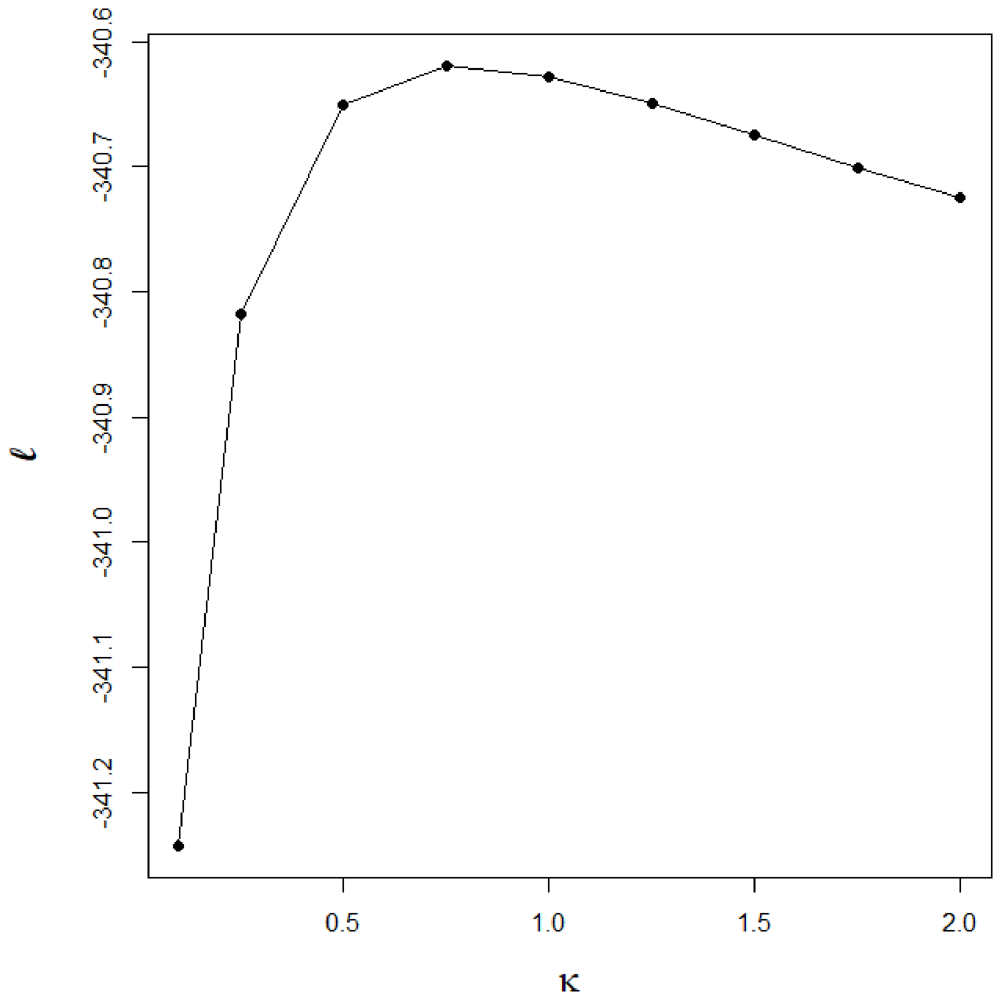

Supplementary Figure 1 Likelihood profile for the κ parameter for the variogram of wheat Zn concentration

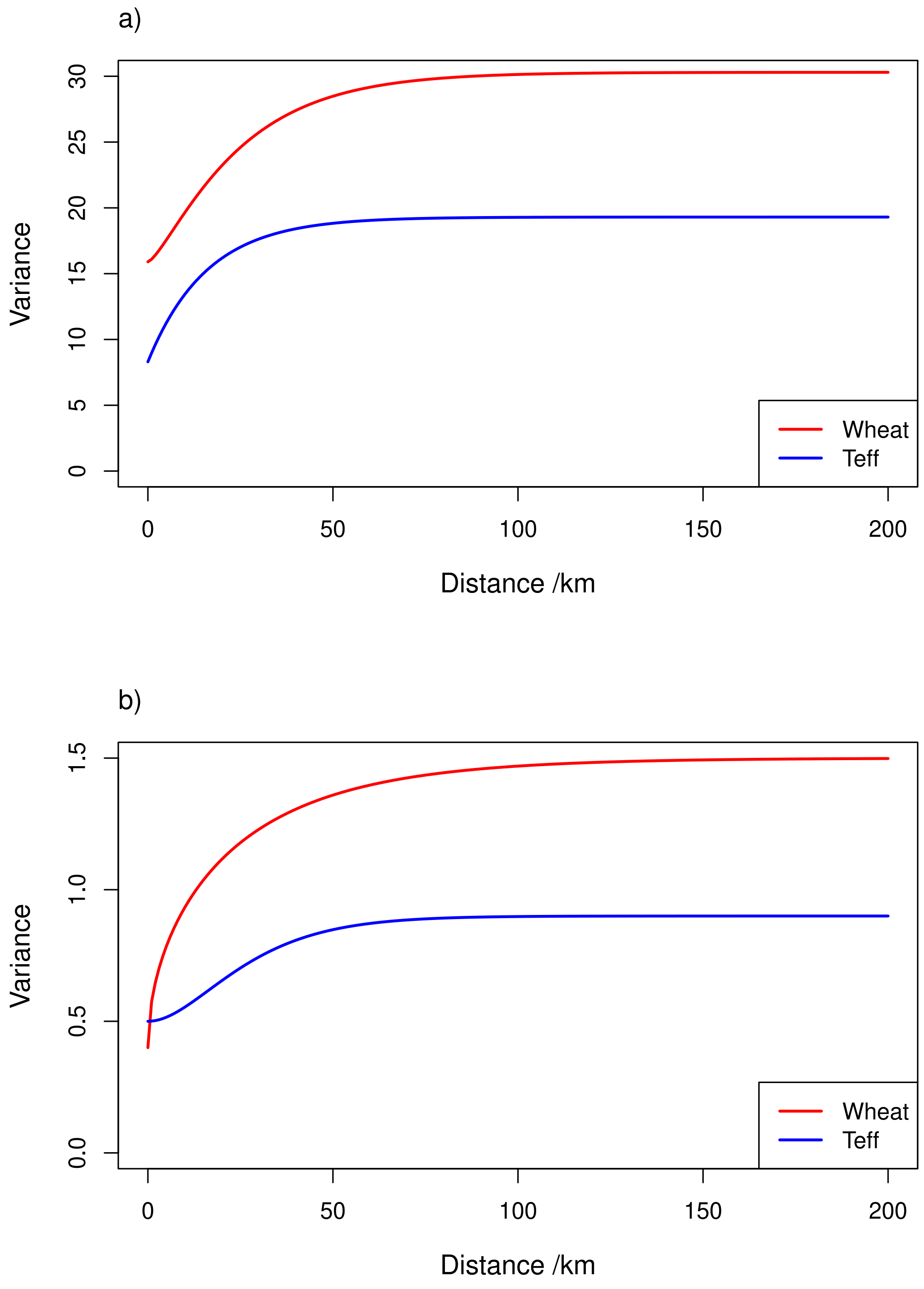

Supplementary Figure 2 Variograms for (a) Zn and (b) Se concentration in wheat and teff grain
